# Supplementary material for: Schizosaccharomyces versatilis represents a distinct evolutionary lineage of fission yeast
Source: Yeast. Author manuscript; Available in PMC 2026 Aug 17. (PMC7619371; doi:10.1002/yea.3919)
Supplement: Supplementary file 1 [file EMS217632-supplement-Supplementary_data.docx]

**Supplementary Data**

Supplementary Figure S1

Calibration of CBS5679 - *S. japonicus* split using (A) RelTime Maximum Likelihood and (B) RelTime Branch Lengths. The node numbers represent divergence times in millions of years. In the trees, the previously-calculated timing of the *S. octosporus – S. osmophilus* split (15.7 MYA) was removed and recalculated along with the *S. japonicus –* CBS56679 split, confirming our previously calculated divergence time of 25 MYA.

Supplementary Figure S2

Long-read (ONT) coverage across translocations on (A) contig13 and (B) contig26, using the IGV genome browser. The solid red line represents the translocations (relative to *S. japonicus* chromosomes 1 and 2), the green squares highlight the flanking BUSCO orthologs, and the grey horizontal bars in the ‘ONT long reads’ panel represent individual ONT reads across the translocations.

Supplementary Table S1

Co-ordinates of the BUSCO single-copy orthologs present in both CBS5679 and *S. japonicus* EI 1.0. The table is sorted by the co-ordinates of orthologs for the chromosome-scale assembly *S. japonicus* EI 1.0.

Supplementary Table S2

The number of Illumina short-read polishing rounds for polishing of CBS5679 and the number changes made for each round

Supplementary Data S1 (s_versatilis.gff.tar.gz)

This archive contains four annotation files in GFF format:

s_versatilis_liftoff.gff – Genome annotation from Ensembl Fungi assembly of *S. japonicus* using Liftoff (Shumate & Salzberg, 2020).

s_versatilis_repeats.gff – Annotation of repeat content from RepeatModeler v2.0.3 (Flynn et al., 2020) and RepeatMasker (v4.1.2-p1) (Smit et al., 2015; Tarailo-Graovac & Chen, 2009).

s_versatilis_trnascan.gff – Annotation of tRNA loci using tRNAscan-SE (v2.0.12) (Chan et al., 2021)

s_versatilis_barrnap_rrna.gff – Annotation of rRNA loci using barrnap (v0.9) (Seeman, 2018).
